# ASPP 092, a phenolic diarylheptanoid from *Curcuma comosa* suppresses experimentally-induced inflammatory ear edema in mice

**DOI:** 10.1016/j.sjbs.2021.06.056

**Published:** 2021-06-24

**Authors:** Aporn Chuncharunee, Poonyawee Khosuk, Rajitpan Naovarat, Feroze Kaliyadan, Gopinathan Pillai Sreekanth

**Affiliations:** a Department of Anatomy, Faculty of Medicine Siriraj Hospital, Mahidol University, Bangkok, Thailand; b Department of Dermatology, King Faisal University, Kingdom of Saudi Arabia; c Siriraj Center of Research Excellence for Molecular Medicine, Faculty of Medicine Siriraj Hospital, Mahidol University, Bangkok, Thailand

**Keywords:** ASPP 092, *Curcuma comosa*, Cyclooxygenase-2, Ear edema, Ethyl phenylpropiolate, Inflammation

## Abstract

*Curcuma comosa* Roxb., family Zingiberaceae, exhibits diverse biological activities. This study was aimed to investigate the anti-inflammatory potential of a major phenolic diarylheptanoid isolated from *C. comosa*, ASPP 092 [(3S)-1-(3,4-dihydroxy-phenyl)-7-phenyl-(6E)-6-hepten-3-ol] in an experimentally-induced inflammatory ear edema model in mice. Ear edema in the mice was induced by the topical application of irritant, ethyl phenylpropiolate (EPP). The topical application of ASPP 092 at the edema site was directed immediately after the EPP application. The edematous responses were assessed at different time points by measuring the thickness of each ear before and after the EPP application followed by histopathology analysis. The expressions of major inflammatory cytokines were analyzed by real-time RT-PCR followed by the immunohistochemistry analysis of cyclooxygenase (COX-2). The topical application of ASPP 092 effectively suppressed the EPP-induced edematous formation in the ear of mice. Histopathological analysis showed substantial improvements in epidermal hyperplasia and inflammatory cell infiltration. ASPP 092 treatment also modulated the expressions of inflammatory cytokines including Tumor Necrosis Factor-α (TNF-α), interleukin-6 (IL-6), interleukin-10 (IL-10), interleukin-1β (IL-1β), and Matrix metalloproteinase-13 (MMP-13). The expressions of cyclooxygenases (COX) including COX-1 and COX-2 were significantly reduced by ASPP 092 treatment. For the first time, our results suggest the efficacy of ASPP 092 to suppress experimentally-induced inflammation in a preclinical model in mice; however, a more detailed evaluation of its mechanism of action is necessary before evaluating its efficacy and safety in randomized trials.

## 1 Introduction

The skin is the largest tissue of the human body and is one of the most active tissues, that is continuously regenerating itself. In addition, exposure to various chemical, physical, and biological factors can lead to different forms of skin damage (Dainichi et al., 2014). Inflammation is the primary response in the immunological defense mechanism to tissue injuries, pathogens, allergens, and other deadly stimuli (Lawrence et al., 2002, Serhan, 2010). The classical symptoms of inflammation include redness, swelling, heat, pain, and loss of function (Miller et al., 2018, Spector and Willoughby, 1963). The symptoms mirror associated cellular changes involving an increase in vascular permeability, migration of leukocytes, chemotaxis, phagocytosis, and inflammation-associated cytokine responses (Karsdal et al., 2013, Vane and Botting, 1995).

The topical application of ethyl phenylpropiolate (EPP) was previously used to induce inflammation in the ears of experimental rats (Farah and Samuelsson, 1992) and further used to investigate the efficacy of prospective therapeutics in inflammatory responses (Intahphuak et al., 2010, Nualkaew et al., 2009). Prostaglandins are important inflammatory mediators and the cyclooxygenase-2 (COX-2) pathway is mainly responsible for the production of prostaglandins involved in inflammation (Seibert and Masferrer, 1994). Phenylbutazone, a nonsteroidal anti-inflammatory drug (NSAID) has been reported to inhibit the expression of COX-2 (Beretta et al., 2005) and was previously used as a reference drug in the treatment of EPP-induced ear edema (Sireeratawong et al., 2012a, Sireeratawong et al., 2012b).

*Curcuma comosa Roxb*. (*C. comosa*) is an indigenous plant in the Zingiberaceae family, which has long been used in Thai traditional medicine for the treatment of inflammation in postpartum uterine bleeding. The estrogenic activity of *C. comosa* has been extensively characterized in both in vitro and in vivo models (Winuthayanon et al., 2013, Winuthayanon et al., 2009a, Winuthayanon et al., 2009b). Diarylheptanoids of both nonphenolic and phenolic have been identified as the major components in the *C. comosa* extract (Suksamrarn et al., 2008, Winuthayanon et al., 2009a). One of the major phenolic diarylheptanoids of *C. comosa*, ASPP 092 [(3S)-1-(3,4-dihydroxy-phenyl)-7-phenyl-(6E)-6-hepten-3-ol] was previously identified for its anti-oxidant properties, which was comparable with Vitamin C and the water-soluble Vitamin E analog, Trolox (Jariyawat et al., 2009). In phorbol-12-myristate-13-acetate (PMA)-stimulated PBMC and U937 cells, ASPP 092 was able to restrict the release of pro-inflammatory cytokines like TNF-α and IL-1β, thereby suppressing the expression of IkB kinase and activation of nuclear factor-kB (Sodsai et al., 2007). However, as of now, the therapeutic possibilities of *C. comosa* and its diarylheptanoids have mostly been investigated in the in vitro conditions, and further studies are required for conclusive evidence of effectiveness. The present study was aimed to investigate the topical anti-inflammatory effect of ASPP 092 in acute skin inflammation in mice.

## 2 Experimental section

### 2.1 Chemicals

**Fig. 1:**
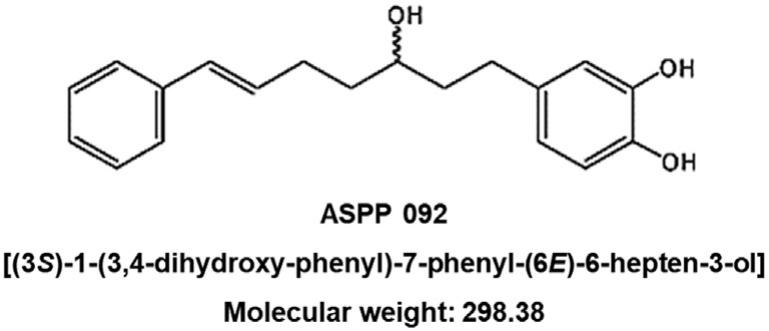
**Chemical structure of diarylheptanoid isolated from *Curcuma comosa*, ASPP 092,** [(3S)-1-(3,4-dihydroxy-phenyl)-7-phenyl-(6E)-6-hepten-3-ol], Molecular weight: 298.38.

Rhizomes of *C. comosa* were purchased from Nakhorn Pathom Province, Thailand (Voucher Number: SCMU No. 300). ASPP 092, Diarylheptanoid [(3S)-1-(3,4-dihydroxy-phenyl)-7-phenyl-(6E)-6-hepten-3-ol] with molecular weight: 298.38, (Fig. 1) was isolated from the *C. comosa* as previously described (Suksamrarn et al., 2008). The purity of the compound used in the study was > 98% determined by HPLC. Ethyl phenylpropiolate (EPP) and phenylbutazone were purchased from Fluka Chemicals Co., Ltd., Japan, and Sigma Aldrich Chem. Co. (Sigma, St. Louis, USA), respectively. All chemicals and reagents used in this study were of analytical grade.

### 2.2 Animals, induction of ear edema and treatments

Adult male ICR mice (weighing 30–35 g) were obtained from the National Laboratory Animal Center, Mahidol University, Salaya Campus, Nakornpathom Province, Thailand. All animals were kept in a controlled room on a 12:12 h light–dark cycle, temperature (25 °C ± 2 °C), relative humidity (65%±5%), allowed free access to water and standard diet (C.P. Mice feed, S.W.T. co., Samutprakan, Thailand), and acclimatized the laboratory facility for 1 week before the experiment. The experimental protocol was approved by the Siriraj Animal Care and Use Committee (Si-ACUC), Faculty of Medicine Siriraj Hospital, Mahidol University, Bangkok, Thailand (SI-ACUP-005/2554 and SI-ACUP-011/2555) under the guidelines of the National Research Council (NRC), Thailand.

The topical application of ethyl phenylpropiolate (EPP) at a dosage of 1 mg/ear dissolved in acetone (20 μL) was applied to the inner and outer surfaces of the ear skin to induce ear edema in mice. Further, they were randomly assigned into 5 groups (N = 8 per group) as follows: mice receiving a vehicle as a control; receiving phenylbutazone 1 mg/ear dissolved in acetone (20 μL) as a positive control and a third group receiving ASPP 092 treatment at three different dosages (0.5, 1.0 or 2.0 mg) dissolved in acetone (20 μL). The gross appearance of the edema site was recorded at different intervals − 0, 15, 30, 60, and 120-minute post-treatment using a Samsung S860 digital camera.

### 2.3 Evaluation of ear edema inhibition

Ear edema was quantified as the increase in the ear thickness in mice. The thickness of the ear edema was measured near the tip of the ear just distal to the cartilaginous ridge, with a digital vernier caliper (Oudi®, Japan) before and 15, 30, 60, 120 min after application of EPP. A single investigator conducted all the ear measurements to reduce experimental error. The increase in the ear thickness was compared with that of the control group and the percentage of inhibition of anti-inflammatory EPP-induced mouse ear edema was calculated with the following formula:

$$\%\;\text{EDI}=\left(\text{EDc}-\text{EDt}\right)/\text{EDc x}\;100$$

EDc = Ear thickness after EPP treatment in the control group at time points 0, 15, 30, 60, or 120 min.

EDt = Ear thickness after EPP treatment in the test group at corresponding time points 0, 15, 30, 60, or 120 min.

% EDI = Percentage of mouse ear edema inhibition of the test group at corresponding time points 0,15,30,60, or 120 min.

### 2.4 Histopathology analysis

All animals were sacrificed under anesthesia by an overdose of pentobarbital sodium (Nembutal®) 2 h post-treatment. Ear specimens were removed, fixed in 10% formalin for at least 24 h, washed with distilled water 1 h, dehydrated with alcohol, cleared in xylene, embedded in paraffin, sectioned, and stained with hematoxylin and eosin. Each mouse-ear section was evaluated under a light microscope (LM) equipped with a digital camera. In the mouse ear, the entire sections including the inner and outer sides were evaluated for the changes in the epidermis and dermis. The epidermal parameters evaluated included - sub-epidermal micro blisters formation, ulceration, necrosis, and crust formation. Dermal parameters evaluated included- hemorrhage, acute inflammation, and necrosis.

### 2.5 Realtime RT-PCR

The total RNA from the ear tissue samples was isolated using the RNeasy Mini Kit (Qiagen, CA, USA). An equal amount of the RNA samples (N = 6) from the individual group of mice were reverse-transcribed to cDNA using the first-strand cDNA Synthesis Kit (Qiagen, CA, USA). The cDNA samples were then amplified by quantitative real-time PCR. The mRNA expressions of inflammatory cytokines including Tumor Necrosis Factor-α (TNF-α), interleukin-6 (IL-6), interleukin-10 (IL-10), interleukin-1β (IL-1β), Matrix metalloproteinase-13 (MMP-13), cycloxygenase-1 (COX-1) and cycloxygenase-2 (COX-2) were analyzed using the primer designs (TNF-α F: 5′- AGC CCC CAG TCT GTA TCC TT −3′, TNF-α R: 5′- CTC CCT TTG CAG AAC TCA GG −3′, IL-1β F: 5′- GCC CAT CCT CTG TGA CTC AT −3′, IL-1β R: 5′- AGG CCA CAG GTA TTTT GTC G −3′, MMP13 F: 5′- ATC CTG GCC ACC TTC TTC TT −3′, MMP13 R: 5′- TTT CTC GGA GCC TGT CAA CT −3′, TGF-β1F: 5′- TTG CTT CAG CTC CAC AGA GA −3′, TGF-β1 R: 5′- TGG TTG TAG AGG GCA AGG AC −3′, IL-10F: 5′- CCA AGC CTT ATC GGA AAT GA −3′, IL-10 R: 5′- TTT TCA CAG GGG AGA AAT CG −3′, COX-1F: 5′- AGG AGA TGG CTG CTG AGT TGG −3′, COX-1 R: 5′- AAT CTG ACT TTC TGA GTT GCC −3′, COX-2F: 5′- ACA CAC TCT ATC ACT GGC ACC −3′, COX-2 R: 5′- TTC AGG GAG AAG CGT TTG C −3′ and β-Actin F: 5′- AGC CAT GTA CGT AGC CAT CC −3′, β-Actin R: 5′- CTC TCA GCT GTG GTG GTG AA −3′) and the SYBR Green Real-Time PCR master-mix in an Applied Biosystems® StepOne™ Real-time PCR machine. The Ct values of the gene of interest were normalized to that of the Ct value of the housekeeping gene (β-Actin). The data were analyzed using the 2^−ΔΔC^*t* method.

### 2.6 Immunohistochemistry staining

Ear specimens were harvested from the mice and fixed in 10% formalin for at least 24 h, washed with distilled water 1 h, dehydrated with alcohol, cleared in xylene, embedded in paraffin, sectioned. The paraffin sections were deparaffinized, rehydrated, immersed with 10 mM sodium citrate buffer, pH 6.0 in microwave oven 15 min for antigen unmasking then blocked the endogenous peroxidase activity and nonspecific reaction for 5 min. Tissue sections were incubated in (1:100) Cycloxygenase-2 (COX-2) primary antibody solution in a humidified chamber at 4 °C overnight, washed with PBS and then incubated for 1 h with the secondary antibody solution (1:1 Envision+/ HRP anti-rabbit antibody; Dako Laboratories), washed with PBS and slides were re-incubated in a solution of 0.1 M 3,3′-diaminobenzidine (DAB) in 0.05 M TBS with 0.5 ml 3% H_2_O_2_ DAB solution (Dako Laboratories) for 5 min. Slides were counterstained with hematoxylin. The expression of COX-2 was stained brownish.

### 2.7 Statistical analysis

Data were expressed means + SEMs. The statistical analysis among different groups was conducted by using a one-way analysis of variance (one-way ANOVA) followed by a Bonferroni test for the differences in pairs of means. A p-value of less than 0.05 was considered significant.

## 3 Results

### 3.1 Effect of ASPP 092 on EPP-induced ear edema

**Fig. 2:**
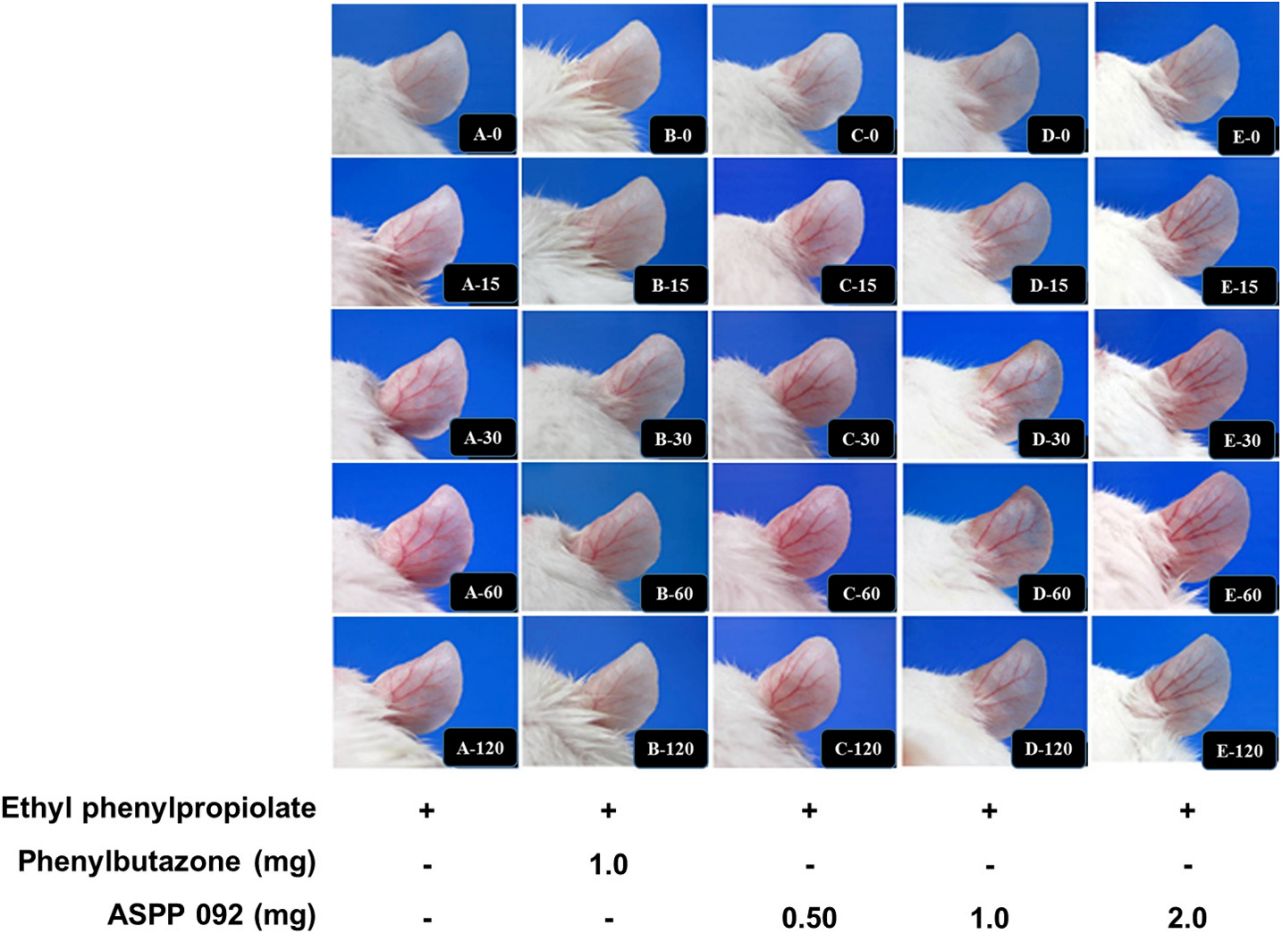
**Effect of ASPP 092 treatment in the gross appearance of EPP-induced ear edema in mice**. The topical application of ethyl phenylpropiolate (EPP) was used to induce ear-edema in the experimental mice. These mice were given treatments with different dosages of ASPP 092 (0.50 mg or 1.0 mg or 2.0 mg, dissolved in 20 µL acetone) or with phenylbutazone (Dosage: 1 mg dissolved in 20 µL acetone) at the same site. The gross appearance of the edema site at different intervals including 0, 15, 30, 60, and 120-minute post-treatment and is represented. A-E in the figure represents different groups of mice and the numbers with it represent respective time points.

In the EPP-induced ear edema mice, the gross appearance of the edema site, and the inflammatory responses which include swelling and redness are shown in Fig. 2. The extent of ear swelling and redness was found to be greatest at 60 min and was maintained at 120 min. Treatment with phenylbutazone substantially inhibited the swelling and redness compared to the control at the corresponding time. Interestingly, treatments with different amounts of ASPP 092 were able to restrict EPP-induced swelling and redness in the ears of mice. Our finding suggests the ability of ASPP 092 to moderate the viable signs of ear edema formation in experimental mice.

### 3.2 Effect of ASPP 092 on cutaneous inflammation in EPP-induced ear edema

**Fig. 3:**
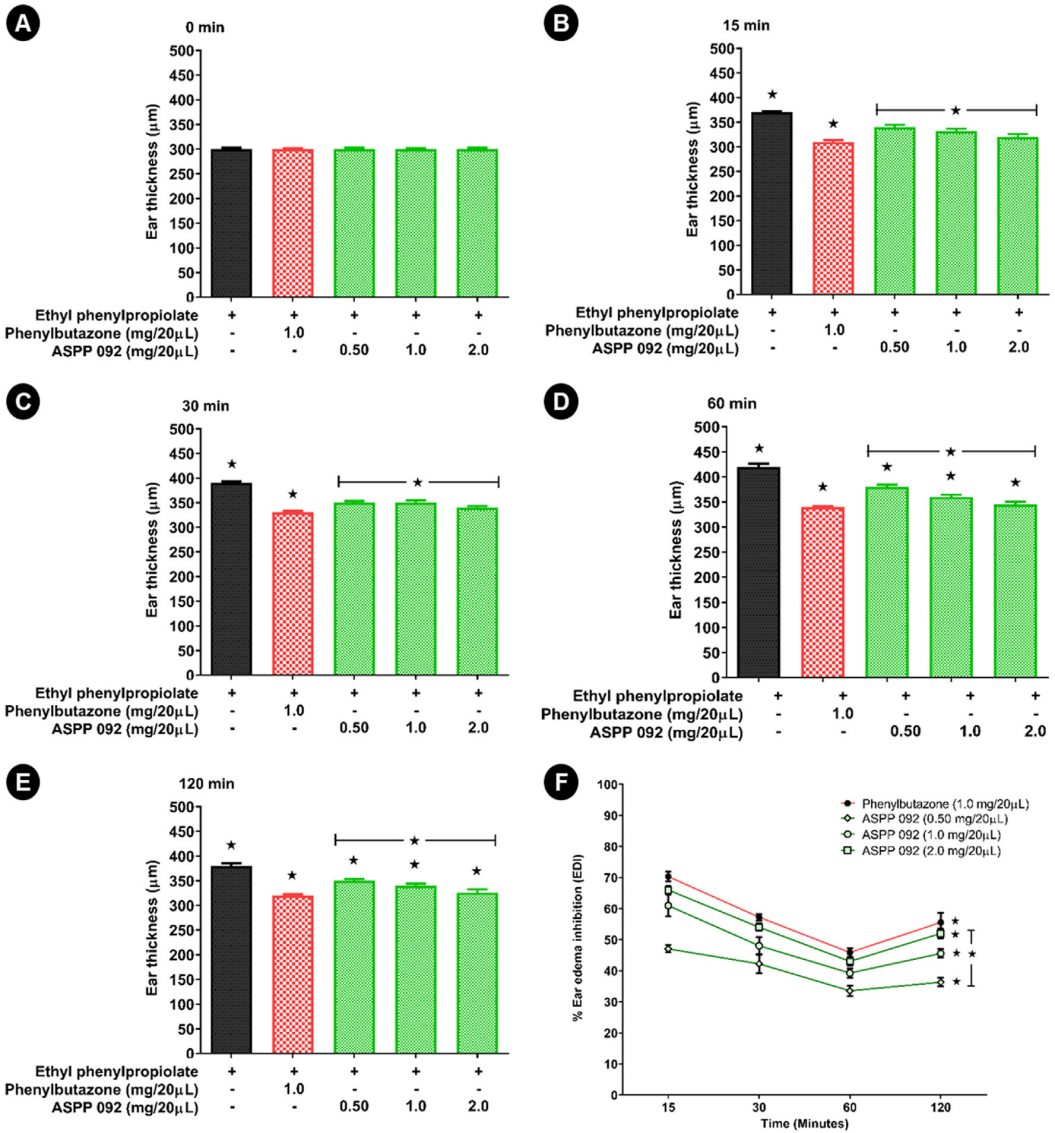
**ASPP 092 treatment suppressed the EPP-induced ear edema in mice.** The topical application of ethyl phenylpropiolate (EPP) was used to induce ear-edema in the experimental mice. These mice were given treatments with different dosages of ASPP 092 (0.50 mg or 1.0 mg or 2.0 mg, dissolved in 20 µL acetone) or with phenylbutazone (Dosage: 1 mg dissolved in 20 µL acetone) at the same site. The ear thickness (ED) at different time points including (A) 0 min (B) 15 min (C) 30 min (D) 60 min and (E) 120 min, and (F) the percentage of ear edema inhibition by the treatment groups compared to that of untreated group was calculated using the same ear thickness values obtained and plotted as a line graph.

Ear thickness or ear edema thickness (ED) of individual mice was measured before (0 min) and after EPP application to evaluate the cutaneous inflammation. In the beginning, the ear thickness was approximately 300 µm (Fig. 3A). The evaluation was continued at various time points which are 15, 30, 60, and 120 min post-EPP, as shown in Fig. 3B, C, D, and E respectively. The application of EPP induced a gradual increase in ear thickness with time. The ear swelling peaked at 60 min, however, thereafter the swellings gradually decreased till 120 min, at the end of the experiment (Fig. 3E). Treatment with ASPP 092 inhibited swelling and reduced the ear thickness at all the time points of the study, compared to that of the untreated EPP-induced ear edema at the corresponding time. These results were similar to those of phenylbutazone-treated mice; however, the potency of phenylbutazone (1 mg) was comparable to that of ASPP 092 treated at 2 mg. In comparison, the ear thickness (ED) in the EPP-induced ear edema mice between the treatment groups and a vehicle group, the percentage of ear edema inhibition (% EDI) was highest at a higher dosage of ASPP 092 treatment which was comparable to that of phenylbutazone (Fig. 3F) at all corresponding time points.

### 3.3 Effect of ASPP 092 on skin histopathology in the EPP-induced ear edema

**Fig. 4:**
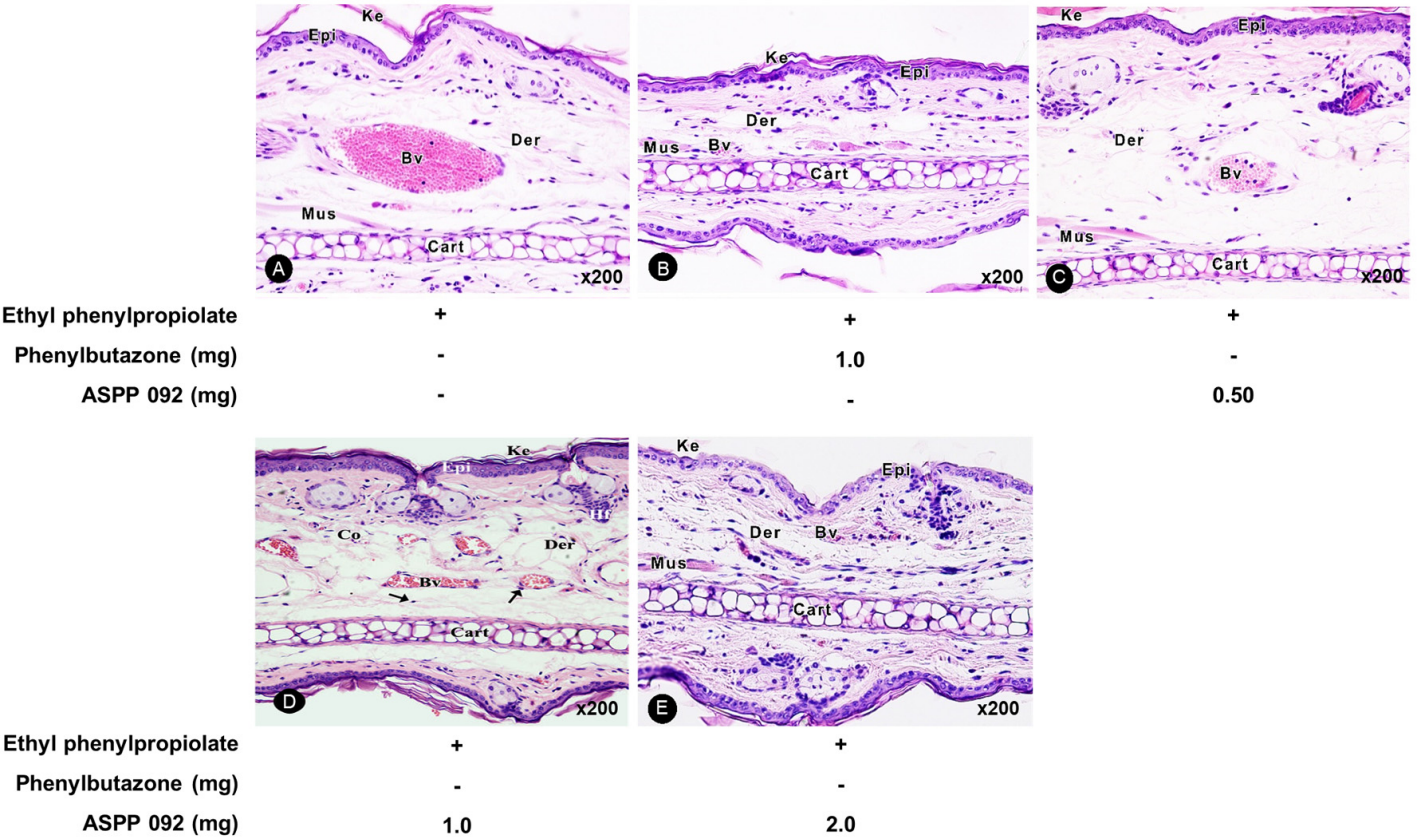
**ASPP 092 treatment improved the histopathology of EPP-induced edema in the ear of mice.** EPP-induced ear edema mice were given treatments with different dosages of ASPP 092 (0.50 mg or 1.0 mg or 2.0 mg, dissolved in 20 µL acetone) or with phenylbutazone (Dosage: 1 mg dissolved in 20 µL acetone) at the same site. The ear of the mice was harvested at 120 min post-treatment and undergone H&E staining and is represented at a magnification of x200. (Arrow: inflammatory cells, Bv; blood vessels, Cart: cartilage, Ke: keratin, Epi: epidermis, Der: dermis and Mus: muscle).

Microscopic analysis of the histological sections of ears at 120 min after the topical application of EPP onto the ear lobes of mice is shown in Fig. 4. The extensive edema formation led to an increase in the dermal thickness which was accompanied by loosening of connective tissue (i.e. fibroblasts, collagen), congestion of vessels, and disorganization of the fibers from the extracellular matrix. The adherence of the inflammatory cells such as mast cells, neutrophils, lymphocytes, eosinophils to the lumina of the blood vessels was observed. The underlying edematous dermis and skeletal muscle tissue contained scattered mixed inflammatory cells (Fig. 4A). Phenylbutazone treatment significantly inhibited these signs of inflammation (Fig. 4B). The effect of different dosages of ASPP 092 including 0.5 mg (Fig. 4C), 1 mg (Fig. 4D), and 2 mg (Fig. 4E) was tested for their efficacy and interestingly, a clear inhibition in the signs of inflammation was observed at a dosage of 2 mg (Fig. 4E). In the dermis, the inflammatory responses were comparably lower; with a comparatively decreased dermis thickness. Signs of milder hemorrhage were observed, with less significant vasodilation, in contrast to that induced by EPP. Moreover, the infiltration of inflammatory cells in the dermis was less intensive. A much lesser accumulation of inflammatory cells in the blood vessel lumina was observed (Fig. 4). ASPP 092 treatment led to improved epidermal architecture; exhibiting reduced thickness and these findings were comparable to that of the improvements seen in the EPP-induced ear edema mice that were treated with phenylbutazone.

### 3.4 Effect of ASPP 092 on the expressions of inflammatory cytokines in the EPP-induced ear edema

**Fig. 5:**
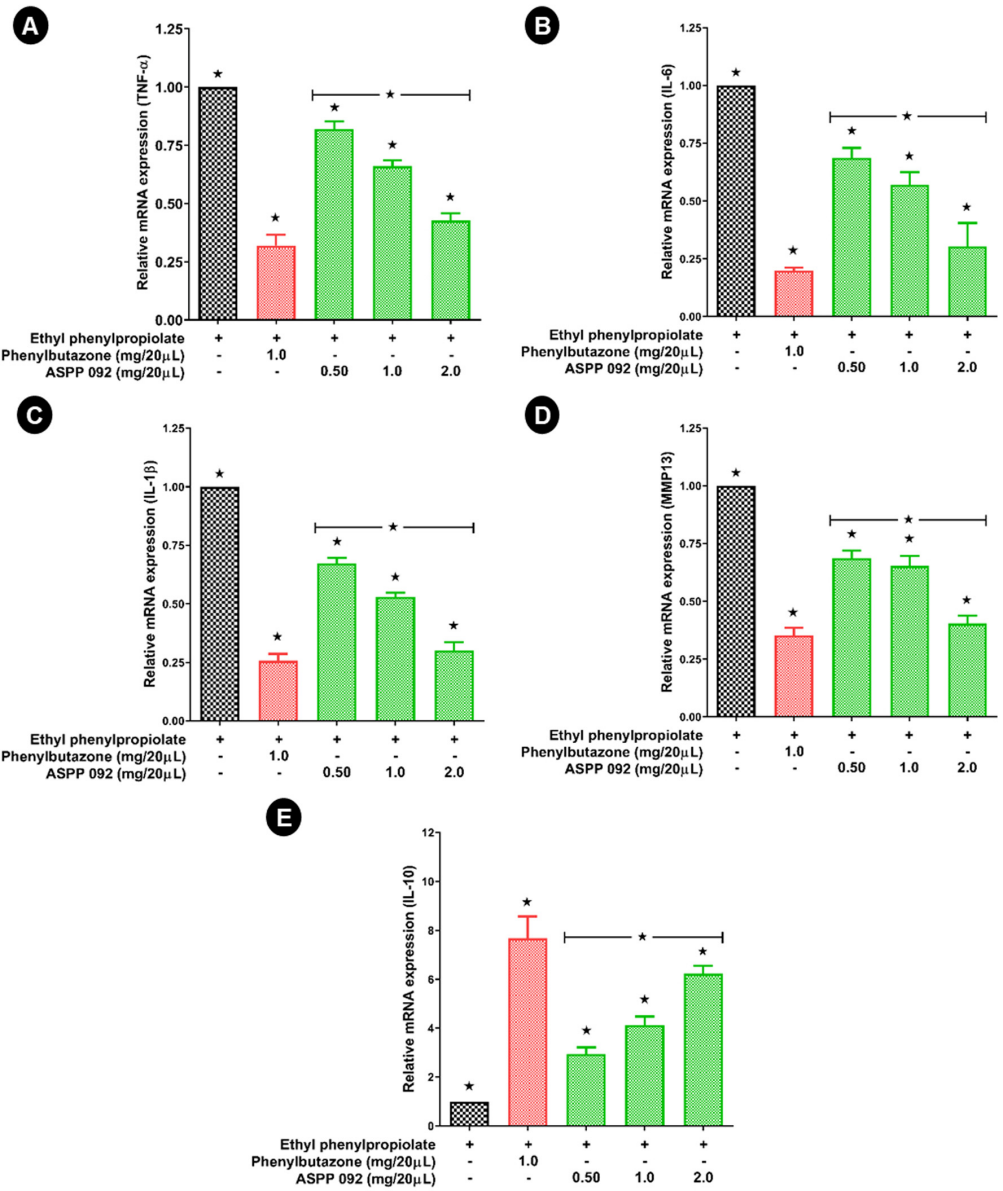
**ASPP 092 treatment modulated the cytokine expressions in EPP-induced ear edema mice.** EPP-induced ear edema mice were given treatments with different dosages of ASPP 092 (0.50 mg or 1.0 mg or 2.0 mg, dissolved in 20 µL acetone) or with phenylbutazone (Dosage: 1 mg dissolved in 20 µL acetone) at the same site. The ear of the mice was harvested at 120 min post-treatment and the mRNA expression of inflammatory cytokines from the ear tissues was estimated using real-time RT-PCR analysis. The expressions of (A) TNF-α (B) IL-6 (C) IL-1β (D) MMP-13 and (E) IL-10 are represented.

The mRNA expression of inflammatory cytokines including TNF-α, IL-6, and IL-10, IL-1β, and MMP-13 was analyzed by RT-PCR. The expression of the genes of interest was normalized using the house-keeping gene, β-actin. Our results suggest a reduced expression of pro-inflammatory cytokines including TNF-α (Fig. 5A), IL-6 (Fig. 5B), IL-1β (Fig. 5C), and MMP-13 (Fig. 5D) in the ASPP 092 treated ear of the mice in a dose-dependent manner when compared to that of the EPP-induced ear edema mice those are untreated. Similarly, reduced expression of these pro-inflammatory cytokines was observed when EPP-educed ear edema mice were treated with phenylbutazone. The expression of the anti-inflammatory cytokine, IL-10 (Fig. 5E) was found higher in ASPP 092-treated mice dose-dependently, compared to that of the ears of an untreated group of mice (those were only applied with EPP). Phenylbutazone-treated ears of the mice have also exhibited a higher expression of IL-10 compared to that of the control group. Our results suggest the efficacy of ASPP 092 in controlling the inflammatory cytokine expressions of the ear of EPP-induced ear edema mice.

### 3.5 Effect of ASPP 092 on cycloxygenase (COX) expression in the EPP-induced ear edema

**Fig. 6:**
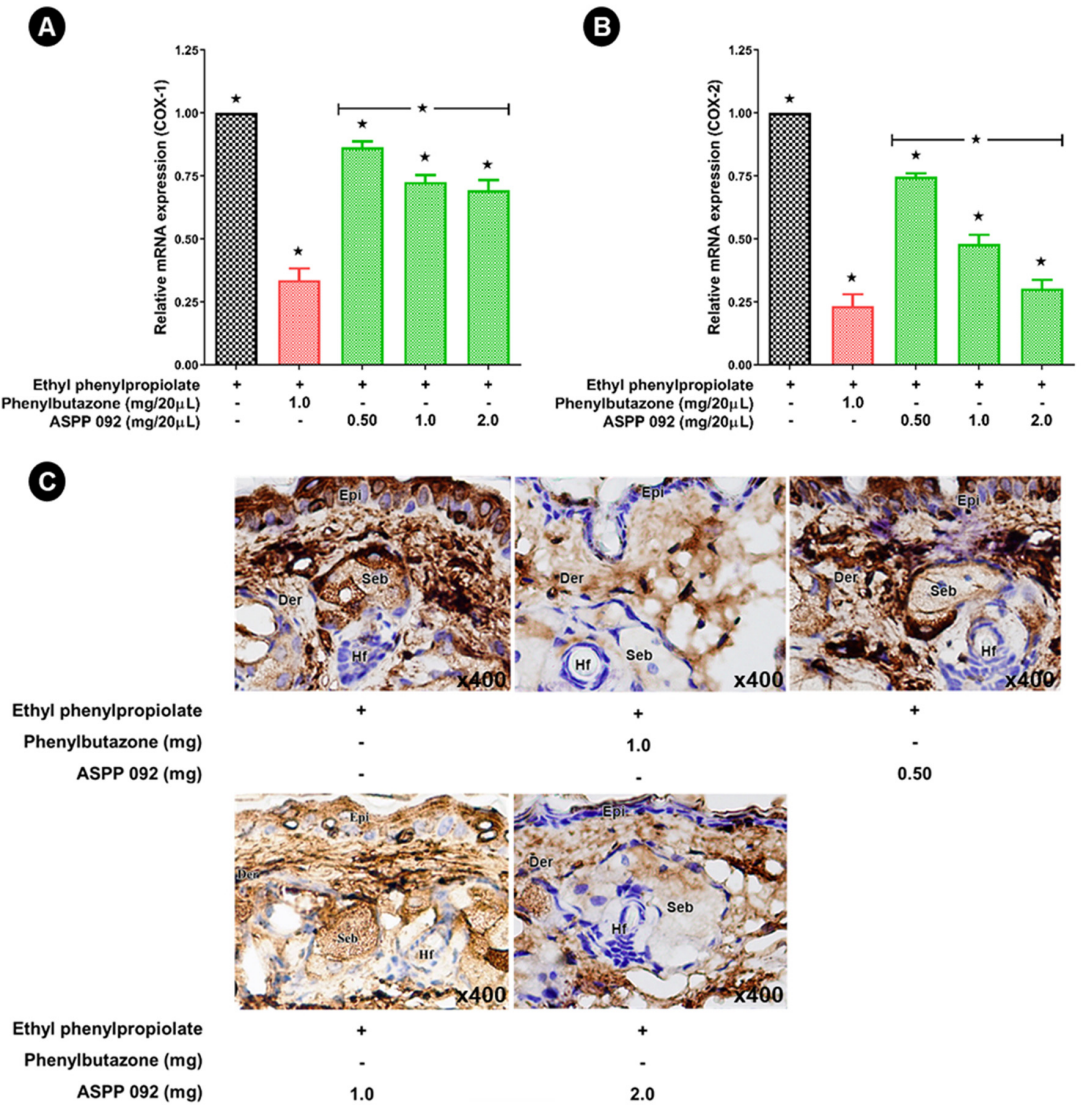
**ASPP 092 treatment reduced the cyclooxygenase (COX) expression in EPP-induced edema in mice.** EPP-induced ear edema mice were given treatments with different dosages of ASPP 092 or phenylbutazone at the same site. The ear of the mice was harvested at 120 min post-treatment. The tissue samples were used to investigate the COX-1 and COX-2 expressions by RT-PCR analysis. The expressions of (A) COX-1 and (B) COX-2 by RT-PCR are shown. (C) Immunohistochemistry analysis was conducted with an anti-COX-2 antibody and counterstained with hematoxylin. The results for each group of mice are represented at a magnification of x400. (Epi: epidermis, De: dermis, Hf: hair follicle, Seb: sebaceous gland).

The mRNA expressions of COX-1 and COX-2 were investigated to further characterize the anti-inflammatory effects of ASPP 092. Reduced expressions of both COX-1 (Fig. 6A) and COX-2 (Fig. 6B) were observed when EPP-induced ear edema mice were treated with ASPP 092; however, the expression of COX-2 was more prominently reduced compared to that of COX-1. The effect of phenylbutazone on the COX-1 expression was better than the ASPP 092 treatment in the ear edema mice, but interestingly the expression of COX-2 was found to be similar in both ASPP 092 and phenylbutazone treated ear edema mice.

**Fig. 7:**
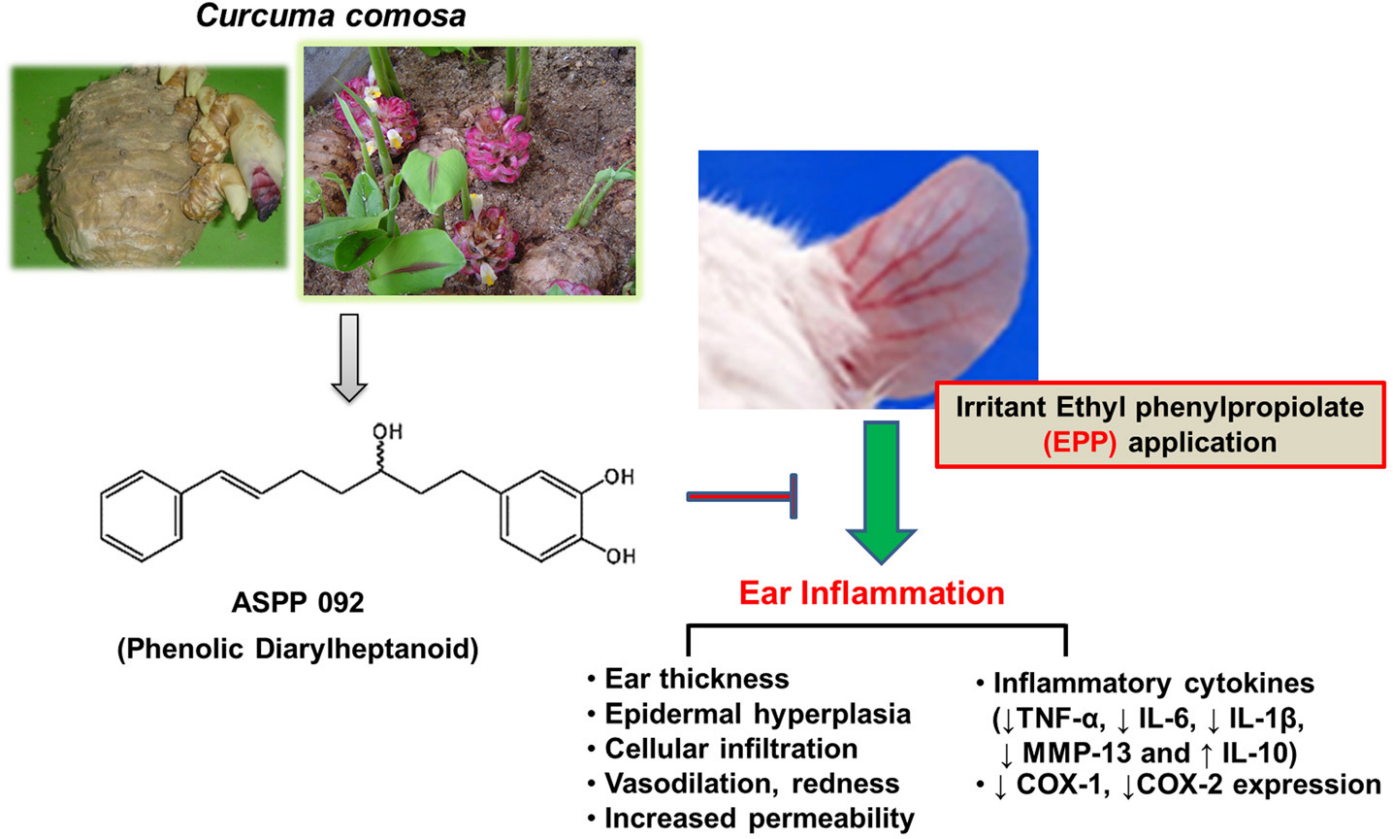
Schematic representation on the effect of ASPP 092 in the inflammatory ear edema model in mice.

Further, immunohistochemistry analysis was conducted to analyze the COX-2 expression in the ears of mice. In EPP-induced ear edema mice, COX-2 expression was extensively high (dark brown peroxidase staining) in the epidermal cells, peripheral cells in the outer root sheath of hair follicles, sebaceous gland, fibroblast-like cells, and capillaries in the dermis (Fig. 6C). In contrast, phenylbutazone-treated mice exhibited a reduced COX-2 expression, especially in the epidermis (Fig. 6C). A dose-dependent lesser extent of cytoplasmic COX-2 was also observed in the ASPP 092 treated mice (Fig. 6C). The high dosage of ASPP 092 (2 mg) exhibited a similar extent to that of phenylbutazone treatment (Fig. 6C), in modulating the COX-2 expression. Our finding suggests the potency of ASPP 092 in reducing the inflammatory responses by reducing the COX-2 expression. A schematic representation of the effect of ASPP 092 on inflammatory ear edema is shown in Fig. 7.

## 4 Discussion

Plant-derived compounds have been used in traditional herbal medicine for treating inflammation (Chao et al., 2010, Kohli et al., 2005). Several diarylheptanoids obtained from different plant sources were previously reported for their pharmacological properties, which were extensively reviewed (Ganapathy et al., 2019). Previously, Curcumin isolated from the *Curcuma longa* was reported for its anti-inflammatory potential in both acute and chronic models of inflammation and its anti-inflammatory efficacy was found to be similar to phenylbutazone (Hewlings and Kalman, 2017, Srimal and Dhawan, 1973). Cassumunarins A, B, and C isolated from the rhizomes of *Zingiber cassumunar* were also exhibited strong anti-inflammatory potential in the 12-O-tetradecanoylphorbol-13-acetate (TPA)-induced ear edema model, and these compounds were found to have stronger anti-inflammatory effects compared to that of curcumin (Masuda et al., 1995). Acerogenin M, a cyclic diarylheptanoid isolated from the methanolic extract of *Acer nikoense* stem bark was found to suppress the TPA-induced ear edema in mice (Akihisa et al., 2006). Three major diarylheptanoids obtained from the *Curcuma xanthorrhiza* were also identified for their anti-inflammatory properties in the mice model of EPP-induced ear edema (Claeson et al., 1996). In the current study, we adopted the EPP-induced ear edema model to investigate the anti-inflammatory potential of ASPP 092, a diarylheptanoid isolated from the C.comosa.

The extract of *C. comosa* was previously reported to prevent bone degradation in mice exhibiting estrogenic deficiency (Weerachayaphorn et al., 2011) and centrilobular necrosis in carbon tetrachloride-induced liver injury in mice (Weerachayaphorn et al., 2010). Two hydroxyl diarylheptanoids isolated from *C. comosa* were identified for their efficacy in moderating oxidative stress-induced cell death in retinal pigment epithelial cells (Jitsanong et al., 2011). Diarylheptanoids including non-phenolic and phenolic (ASPP 001, 047, 049, 091, and 092) were isolated from *C. comosa* and their stereochemistry was extensively characterized (Suksamrarn et al., 2008). DPHD (ASPP 049) accelerated human osteoblast proliferation (Tantikanlayaporn et al., 2013) and facilitated vascular relaxation in the aorta of rats (Intapad et al., 2009). The effect of diarylheptanoids including ASPP 049 and ASPP 092 was studied in hydrogen peroxide (H2O2) exposure-induced oxidative stress in C6 astroglial cells; where ASPP 092 exerted strong cytoprotective effects than that of ASPP 049 (Vattanarongkup et al., 2016). The anti-inflammatory potential of ASPP 092 was previously identified in both PBMC and U937 cells (Sodsai et al., 2007) and the present study identified the efficacy of ASPP 092, suppressing the cutaneous inflammation in an EPP-induced ear edema model in mice.

The ear edema model in animals was extensively used to study inflammation and pain, where inflammatory cytokines contribute to its pathology (Cunha et al., 2005). Different chemical irritants were previously used to induce skin inflammation in the ear of laboratory mice (Blazso and Gabor, 1995, Heo et al., 2018, Rodrigues et al., 2016), including EPP (Pongprayoon et al., 1992). Though the inflammatory patterns differ between irritants; interestingly, the inflammatory response to EPP treatment was found to be exhibited much earlier compared to that of other irritants tested (Patrick et al., 1985). EPP causes instant irritation in the ear of mice, which leads to fluid accumulation and edema characterizing acute inflammatory response; with major symptoms including vasodilation, increased blood flow and vascular permeability, and infiltration of leukocytes that migrate to sites of injury (Patrick et al., 1985). Phenylbutazone, a nonsteroidal anti-inflammatory drug (NSAID) was used as a positive control in the present study; the dosage used was previously reported to reduce the inflammatory symptoms in an ear edema model in mice (Sireeratawong et al., 2012a).

The three dosages of ASPP 092 investigated in the present study exhibited significant inhibitory effects on EPP-induced ear edema at all assessment period (15, 30, 60, and 120 min after EPP application); however, the highest improvements were observed with the highest dosage (2 mg ASPP 092 dissolved in 20 µL acetone). The percentage of ear edema inhibition (% EDI) was at a peak at 15 min after EPP application and gradually decreased until 60 min. A biphasic event of inflammatory responses was reported in the irritant-induced ear edema in animals; where the release of serotonin, histamine, and bradykinin happens in the initial phase (0–1 h) (Sadeghi et al., 2014). In the late phase (after 1 h), because of the neutrophil infiltration into the inflammatory site, inflammatory cytokine responses and various inflammatory mediators contribute to the pathogenesis (Maling et al., 1974, Sadeghi et al., 2013, Santos et al., 2012). In the current study, we studied the late phase and our findings were consistent with the previous studies (Sireeratawong et al., 2013, Zhao et al., 2018). The gross appearance of the ear edema site in the experimental mice was correlated with histological findings to understand the inflammatory pattern. After EPP infiltration into the skin, it stimulates mediators of inflammation including histamine, serotonin, bradykinin, leukotrienes, and prostaglandins (Brattsand et al., 1982) leading to vasodilation, increased vascular permeability, and infiltration of leukocytes (Carlson et al., 1985). These changes impact the edema formation when EPP was applied. Interestingly, our result suggests treatment with ASPP 092 inhibits these cellular pathologies in the EPP applied mice suggesting its potency in moderating the ear edema formation; however, higher effectiveness was observed with the higher dosage of ASPP 092 and phenylbutazone.

Different strategies for the phytoconstituents towards its development to anti-inflammatory drugs were recently reviewed (Patil et al., 2019). Anti-inflammatory activities of compounds show many common chemical mediators and mechanisms of action (Woolf and Max, 2001). The major inflammatory cytokines and their responses on treatment with different natural compounds were reviewed (Azab et al., 2016). One of the major pro-inflammatory cytokines, TNF-α production was regulated by the mononuclear phagocytes and stimulates the macrophages; this leads to induce the secretion of various other inflammatory cytokines including IL-1β and IL-6 (Liao et al., 2013). Our findings suggest the topical application of ASPP 092 could reduce the expressions of pro-inflammatory cytokines TNF-α, IL-6, IL-1β, and MMP-13, but conversely raise the expression of IL-10 in a dose-dependent manner. In a previous study, these cytokine responses were reported to be observed at the late phase of inflammation (Ma et al., 2013) and our results were consistent.

Prostaglandins play a critical role in the generation of the inflammatory response that is triggered after tissue injury. The isoforms of cycloxygenase, both COX-1 and COX-2 was previously reported to mediate the rate-limiting step in the arachidonic acid metabolism (Tanaka et al., 1997). COX-2 is an inducible enzyme that is highly expressed and accountable to release the inflammatory mediator PGE2 during inflammation, further leading to tissue injury (Gandhi et al., 2017, Khan et al., 2007). The COX2-induced prostanoid production was common in inflammatory conditions characterized by edema and tissue injury due to the release of many inflammatory cytokines (Arslan and Zingg, 1996). The higher expressions of TNF-α and IL-1β were reported to initiate a higher COX-2 expression (Park et al., 2004) and our findings were consistent with this study. NSAIDs have been shown to inhibit COX-2 expression and PGE2 generation suggesting their mechanism of action (Wright, 2002). In the present study, ASPP 092 reduced ear thickness with less edema and inhibited the COX2 expression. Interestingly, ASPP 092 at 2 mg essentially elicited the anti-inflammatory effect similar to those of phenylbutazone, an NSAID. Therefore, the anti-inflammatory activity of ASPP 092 may, at least, be related to inhibition of the COX2 enzyme as well as the reduction in the inflammatory signs. Previously, the inhibitors of COX-2 were reported to reduce irritant-induced ear edema in mice (Carlson et al., 1985); however, the topical application of natural compounds was effective to reduce the COX-2 expression, with minimal or no side effects (Attiq et al., 2018). Ginsenoside metabolite compound K was recently reported for its anti-inflammatory properties via down-regulating COX-2 expression (Chen et al., 2019). The diarylheptanoids, oregonin, and hirsutanonol, both were isolated from the bark of *Alnus hirsute* exhibited anti-inflammatory potential by inhibiting the COX-2 expression (Lee et al., 2000). Our findings with ASPP 092 treatment in the EPP-induced ear edema in mice was consistent with these findings.

## 5 Conclusions

Our results demonstrate, for the first time, the topical anti-inflammatory potential of phenolic diarylheptanoid compound, ASPP 092, against acute cutaneous inflammation suggesting to further evaluate its potential as complementary medicine for treating skin disorders. The therapeutic potential of ASPP 092 on local inflammation was, at least, mediated through the inflammatory cytokines and inhibition of the expression of COX-2, however, preclinical evaluations are necessary to identify more details on the mechanism of actions.

## Funding

This research was funded by Mahidol University Grant, grant number R016120002 to G.P.S. and A.C. was supported by the Faculty of Medicine Siriraj Hospital, Mahidol University.
